# RESS has advanced in the CAPES Qualis-Journals system ranking in 2026: we are now classified as A2

**DOI:** 10.1590/S2237-96222026v35e20263001.en

**Published:** 2026-04-07

**Authors:** Everton Nunes da Silva, Jorge Otávio Maia Barreto, Maria Auxiliadora Parreiras Martins, Taís Freire Galvão

**Affiliations:** 1 Universidade de Brasília, Faculdade de Ciências e Tecnologias em Saúde, Brasília, DF, Brazil Universidade de Brasília Faculdade de Ciências e Tecnologias em Saúde Brasília DF Brazil; 2 Fundação Oswaldo Cruz, Gerência Regional de Brasília, Brasília, DF, Brazil Fundação Oswaldo Cruz Gerência Regional de Brasília Brasília DF Brazil; 3 Universidade Federal de Minas Gerais, Faculdade de Farmácia, Belo Horizonte, MG, Brazil Universidade Federal de Minas Gerais Faculdade de Farmácia Belo Horizonte MG Brazil; 4 Universidade Estadual de Campinas, Faculdade de Ciências Farmacêuticas, Campinas, SP, Brazil Universidade Estadual de Campinas Faculdade de Ciências Farmacêuticas Campinas SP Brazil

The publication of the 2021-2024 Qualis-Journals in January 2026 confirmed the consistent and progressive advances made by the *Epidemiologia e Serviços de Saúde: revista do SUS* (RESS) over the years. RESS was first evaluated in this journal classification system during the 2013-2016 four-year evaluation cycle, when the journal achieved the B2 classification in the Public Health evaluation area. In the following four-year period (2017-2020), the journal received the A3 classification, which was applied across all evaluation areas by the Brazilian Federal Agency for Support and Evaluation of Graduate Education (*Coordenação de Aperfeiçoamento de Pessoal de Nível Superior*, CAPES). In the last four-year period (2021-2024), the journal achieved the A2 classification^1^, the second-best position among nine strata (A1, A2, A3, A4, B1, B2, B3, B4, and C). 

In the last four-year cycles, CAPES has refined its journal evaluation criteria based on internationally adopted bibliometric indicators, promoting comparability across fields of knowledge and the reproducibility of the metrics used^2^. Another aspect considered is the indexing and internationalization of journals, which expand the global dissemination of scientific articles^2^.

CAPES adopted two forms of grouping indicators: I) Qualis Reference 1 (QR1), using the percentiles of the CiteScore^3^ and from Clarivate's Journal Citation Reports (JCR)^4^ and, in the absence of these, the Google Scholar h5-index^5^; and ii) Qualis reference 2 (QR2), based on the Google Scholar h-index (h5 or h10)^5^ for defining the percentiles. In both cases, the journal is compared with the others in the evaluation area (both national and international), and its relative position in the percentiles of the bibliometric indicators used determines its stratum in the Qualis-Journal ranking. Each evaluation area establishes the most appropriate grouping method for the journals for which it is considered the leading area (*área-mãe,* in Portuguese). The Public Health evaluation area adopted the QR2, through which RESS was classified. Leading areas could also make additional adjustments to journal strata, provided that such modifications did not exceed 30% of the strata^2^.

Evolving within the Qualis-Journals classification represents a significant achievement for RESS and other Brazilian journals, especially when considering the global comparative context of scientific communication. Internationally, there is an increasing concentration of the scientific publishing market among a small number of large publishers^6^. Most of these publishers follow the article processing charge (APC) model, frequently with high fees, creating challenges for the sustainable dissemination of scientific evidence as a socially relevant public good.

This model can also increase socioeconomic asymmetries by favoring authors affiliated with institutions with greater capacity to raise financial resources^7^. In this context, it is essential to implement public policies aimed at reducing these disparities in funding for national scientific journals^8^.

Since its creation, RESS has followed an open-access model, charging authors no article processing fees, whether at submission or upon publication. In addition, articles are published in both Portuguese and English, without any fees for translation services. These characteristics align with the process of expanding the journal's internationalization through publication in English, while promoting the use of evidence by decision-makers through the availability of content in Portuguese.

At the same time, RESS has been improving its editorial processes and scope, in line with its mission to disseminate scientific knowledge of relevance to the strengthening of the Brazilian Unified Health System (Sistema Único de Saúde, SUS)^9^, targeting health managers and professionals, researchers, and students in Public Health and related fields. These advances are also reflected in the quantity and quality of the articles published, as well as in the journal's external evaluation in the Qualis-Journals system over recent four-year evaluation cycles and in other classification systems.

RESS holds a unique position in the Brazilian scientific communication landscape, establishing itself as one of the leading channels for disseminating scientific evidence to inform decision-making within the SUS. The recognition of its editorial quality represents both an institutional advancement and a reinforcement of its credibility as a qualified space for producing and disseminating scientific knowledge relevant to our health system. This recognition helps to increase the journal's visibility among research institutions and public health organizations, strengthening RESS's capacity to continue contributing to the use of evidence in the formulation, implementation, and evaluation of public health policies in Brazil.

By sharing this achievement with RESS readers, we reaffirm our ethical commitment to the journal's continuous improvement and to prioritizing studies with greater potential to strengthen the SUS. We invite the scientific community and health service professionals to submit their research to RESS to promote the dissemination of evidence that is both necessary and relevant.
