# Supplementary material for: RESS has advanced in the CAPES Qualis-Journals system ranking in 2026: we are now classified as A2
Source: Epidemiol Serv Saude. 2026 Apr 7;35:e20263001. doi: 10.1590/S2237-96222026v35e20263001.en (PMC13686294; doi:10.1590/S2237-96222026v35e20263001.en)
Supplement: Translation

# Translation: A RESS avançou na classificação do Qualis Periódicos da CAPES em 2026: agora somos A2

**DOI:** 10.1590/S2237-96222026v35e20263001.pt

**Authors:** Everton Nunes da Silva, Jorge Otávio Maia Barreto, Maria Auxiliadora Parreiras Martins, Taís Freire Galvão

**Affiliations:** 1 Universidade de Brasília, Faculdade de Ciências e Tecnologias em Saúde, Brasília, DF, Brasil Universidade de Brasília Faculdade de Ciências e Tecnologias em Saúde Brasília DF Brasil; 2 Fundação Oswaldo Cruz, Gerência Regional de Brasília, Brasília, DF, Brasil Fundação Oswaldo Cruz Gerência Regional de Brasília Brasília DF Brasil; 3 Universidade Federal de Minas Gerais, Faculdade de Farmácia, Belo Horizonte, MG, Brasil Universidade Federal de Minas Gerais Faculdade de Farmácia Belo Horizonte MG Brasil; 4 Universidade Estadual de Campinas, Faculdade de Ciências Farmacêuticas, Campinas, SP, Brasil Universidade Estadual de Campinas Faculdade de Ciências Farmacêuticas Campinas SP Brasil

A divulgação do novo Qualis Periódicos 2021-2024, em janeiro de 2026, confirmou os avanços consistentes e progressivos alcançados pela *Epidemiologia e Serviços de Saúde: revista do SUS* (RESS) ao longo dos anos. A primeira apreciação da RESS nesse sistema de classificação de periódicos aconteceu no quadriênio 2013-2016, quando a revista obteve a classificação B2 na área de avaliação da Saúde Coletiva. No quadriênio seguinte (2017-2020), obteve a classificação A3, a qual passou a ser aplicada a todas as áreas de avaliação da Coordenação de Aperfeiçoamento de Pessoal de Nível Superior (CAPES). No último quadriênio (2021-2024), alcançou a classificação A2,^1^ a segunda melhor posição em um conjunto de nove estratos (A1, A2, A3, A4, B1, B2, B3, B4 e C). 

O aprimoramento dos critérios de avaliação dos periódicos nos últimos quadriênios, conduzido pela CAPES, baseou-se em indicadores bibliométricos adotados internacionalmente, favorecendo a comparabilidade entre áreas de conhecimento e a reprodutibilidade das métricas adotadas^2^. Outro ponto considerado é a indexação e internacionalização dos periódicos, por meio das quais se ampliam globalmente os meios de disseminação dos artigos científicos^2^.

A CAPES adotou duas formas de agrupamento de indicadores: i) Qualis Referência 1 (QR1), com uso dos percentis da CiteScore^3^ e do Journal Citation Reports (JCR) da Clarivate^4^ e, na ausência destes, o Índice h5 do Google Scholar^5^; e ii) Qualis Referência 2 (QR2), com uso do Índice h (h5 ou h10) do Google Scholar^5^ para definição dos percentis. Nos dois casos, o periódico é comparado com os demais da área (nacionais e estrangeiros), e sua posição relativa nos percentis dos indicadores bibliométricos usados define seu estrato no Qualis Periódico. Cada área de avaliação estabelece a forma de agrupamento mais pertinente aos periódicos em que é considerada área-mãe. A Saúde Coletiva adotou o QR2, pelo qual a RESS foi classificada. Ajustes adicionais nos estratos das revistas foram facultados às áreas-mãe, desde que não ultrapassassem 30% de alterações nos estratos^2^.

Evoluir na classificação do Qualis Periódicos representa uma conquista relevante para a RESS e para outros periódicos brasileiros, especialmente se considerarmos o contexto comparativo global da comunicação científica. No cenário internacional, observa-se crescente concentração do mercado de publicações científicas em um pequeno grupo de grandes editoras^6^. Em sua maioria, essas editoras adotam o modelo de cobrança de taxas de processamento de artigos (*article processing charges*, APC), frequentemente com valores elevados, o que impõe desafios à sustentabilidade da disseminação de evidências científicas como bens públicos de relevância social.

Esse modelo também pode ampliar assimetrias socioeconômicas, ao favorecer autores vinculados a instituições com maior capacidade de captação de recursos financeiros^7^. Nesse contexto, torna-se fundamental a adoção de políticas públicas voltadas à redução dessas disparidades no financiamento de periódicos científicos nacionais^8^.

Desde sua criação, a RESS adota o modelo de acesso aberto, e não cobra dos autores taxas de processamento de artigos, seja na submissão, seja na publicação. Ademais, os artigos são disponibilizados em português e em inglês, sem cobrança de serviço de tradução. Essas características convergem com o processo de ampliação da internacionalização da revista, por meio da publicação em inglês, ao mesmo tempo que favorecem o uso das evidências por tomadores de decisão, ao serem disponibilizados os conteúdos em português.

Paralelamente, a RESS tem aprimorado seus processos editoriais e seu escopo, em consonância com sua missão de difundir conhecimento científico de interesse para o aprimoramento do Sistema Único de Saúde (SUS)^9^, tendo como público-alvo gestores, profissionais de saúde, pesquisadores e estudantes de saúde coletiva e áreas afins. Esses progressos também se refletem na quantidade e na qualidade dos artigos publicados, bem como na avaliação externa obtida pela revista no sistema Qualis Periódicos, nos últimos quadriênios, e em outros sistemas de classificação.

A RESS ocupa uma posição singular no cenário da comunicação científica brasileira, ao atuar como um dos principais veículos de disseminação de evidências científicas voltadas à tomada de decisão no SUS. O reconhecimento da sua qualidade editorial significa não apenas um avanço institucional, mas também um reforço de sua credibilidade como espaço qualificado para a produção e disseminação de conhecimento científico de interesse para nosso sistema de saúde. Esse reconhecimento ajuda a ampliar a visibilidade da revista entre instituições de pesquisa e organizações de saúde pública, fortalecendo sua capacidade de continuar contribuindo para o uso de evidências na formulação, na implementação e na avaliação de políticas de saúde pública no Brasil.

Ao compartilhar essa conquista com os leitores da RESS, reafirmamos nosso compromisso ético com o contínuo aprimoramento da revista e com a priorização de estudos com maior potencial de contribuir para o fortalecimento do SUS. Estimulamos a comunidade científica e os profissionais dos serviços de saúde a submeterem suas pesquisas à RESS, fomentando a disseminação de evidências necessárias e relevantes.
